# Single‐Cell Transcriptomic Profiling Reveals Cellular Heterogeneity and Identifies Novel Therapeutic Targets in Osteosarcoma

**DOI:** 10.1155/ijog/4040246

**Published:** 2026-07-18

**Authors:** Hui Li, Changjiang Sun, Minjie Yang

**Affiliations:** ^1^ Department of Endocrinology, Shaanxi Provincial People′s Hospital, Xi′an City, Shaanxi Province, China; ^2^ Department of Orthopaedics, Yuechi Hospital of T.C.M., Guang′an City, Sichuan Province, China; ^3^ Department of Orthopedics, Jiujiang City Key Laboratory of Cell Therapy, Jiujiang No. 1 People′s Hospital, Jiujiang City, Jiangxi Province, China, jxjjsdyrmyy.cn

**Keywords:** biomarker validation, cellular heterogeneity, osteosarcoma, POSTN, single-cell RNA sequencing, tumor microenvironment

## Abstract

**Background:**

Osteosarcoma is the most prevalent primary malignant bone tumor predominantly affecting children and adolescents, yet prognosis for metastatic disease remains dismal. Understanding the cellular complexity within the tumor microenvironment is essential for developing targeted therapeutic strategies.

**Methods:**

We performed comprehensive single‐cell RNA sequencing analysis on an osteosarcoma tissue sample (GSM4952363) using the Seurat pipeline (v4.3.0). Following rigorous quality control (200–6000 genes per cell, < 15% mitochondrial reads), cells were filtered for downstream analysis. Dimensionality reduction (PCA and UMAP) and unsupervised clustering (Louvain algorithm, resolution = 0.8) identified seven distinct cellular clusters. Differential expression analysis (Wilcoxon rank‐sum test, |log₂FC| > 0.25, adjusted *p* < 0.05) identified cluster‐specific markers, while Gene Ontology and KEGG pathway enrichment analyses (clusterProfiler, adjusted *p* < 0.05) revealed functional programs. Four candidate genes (F11, ACRP2, LEPR, and POSTN) were selected for validation by quantitative real‐time PCR (mRNA level) and ELISA (protein level) in MG‐63 osteosarcoma cells compared to hFOB 1.19 normal osteoblasts.

**Results:**

Single‐cell transcriptomic profiling identified seven distinct cellular clusters within the osteosarcoma microenvironment, including macrophages (Cluster 0, 28.0%), osteoblasts (Cluster 1, 24.2%), fibroblasts (Cluster 2, Fibro_COMP, 14.7%), proliferating cells (Cluster 3, 12.3%), osteoclasts (Cluster 4, 11.6%), monocytes (Cluster 5, 6.3%), and T cells (Cluster 6, 2.9%). Functional enrichment analysis highlighted activation of PI3K‐Akt signaling, focal adhesion, and extracellular matrix organization as core pathways. qRT‐PCR validation (mRNA level) demonstrated that F11 was significantly downregulated (0.31 ± 0.04 vs. 1.00 ± 0.07, 69% reduction, *p* < 0.001), whereas ACRP2 (2.87 ± 0.33‐fold), LEPR (4.52 ± 0.48‐fold), and POSTN (6.23 ± 0.57‐fold) were significantly upregulated (all *p* < 0.001). ELISA validation (protein level) confirmed consistent trends: F11 protein decreased by 65% (0.35 ± 0.05 vs. 1.00 ± 0.08, *p* < 0.001), whereas ACRP2 (2.64 ± 0.29‐fold), LEPR (4.18 ± 0.44‐fold), and POSTN (5.89 ± 0.53‐fold) protein levels were elevated (all *p* < 0.001). Among the four candidates, POSTN exhibited the most pronounced changes at both mRNA and protein levels, suggesting its involvement in osteosarcoma matrix remodeling.

**Conclusions:**

This study provides a single‐cell transcriptomic atlas of the osteosarcoma microenvironment, revealing substantial cellular heterogeneity. The differentially expressed genes F11, ACRP2, LEPR, and POSTN represent candidate biomarkers that warrant further investigation for their potential roles in osteosarcoma biology and as putative therapeutic targets.

## 1. Introduction

Osteosarcoma represents the most prevalent primary malignant bone tumor, predominantly affecting children, adolescents, and young adults, with an annual incidence of approximately three to four cases per million individuals worldwide [1, 2]. Despite advances in multimodal treatment strategies combining surgery, chemotherapy, and radiotherapy, the 5‐year survival rate for patients with metastatic or recurrent osteosarcoma remains below 30% [3–5], highlighting the urgent need for improved understanding of disease mechanisms and development of novel therapeutic approaches. The pathogenesis of osteosarcoma involves complex interactions between genetic predisposition, cellular dysfunction, and microenvironmental factors, yet the precise causal mechanisms underlying disease initiation and progression remain inadequately characterized.

The tumor microenvironment (TME) in osteosarcoma consists of diverse cellular components including malignant osteoblasts, stromal fibroblasts, immune infiltrates, vascular elements, and bone‐resident cell types, all of which engage in complex paracrine and juxtacrine interactions that collectively influence tumor initiation, progression, and therapeutic response [6–8]. Deciphering this cellular complexity is essential for identifying cell type–specific vulnerabilities and developing targeted therapeutic strategies [9, 10].

Recent advances in single‐cell RNA sequencing (scRNA‐seq) technology have revolutionized our understanding of tumor biology by enabling comprehensive characterization of cellular heterogeneity within the TME at unprecedented resolution [11–13]. Osteosarcoma exhibits substantial cellular diversity, comprising malignant osteoblastic cells, stromal fibroblasts, immune cells, vascular components, and other specialized cell types that collectively influence tumor progression, therapeutic response, and clinical outcomes [6]. However, systematic characterization of cellular subpopulations, their gene expression programs, and functional roles in osteosarcoma pathogenesis remains limited. Understanding this cellular complexity is essential for identifying cell type–specific vulnerabilities and developing targeted therapeutic strategies [7, 8].

This study employs single‐cell transcriptomic profiling to comprehensively characterize cellular heterogeneity and molecular signatures in the osteosarcoma microenvironment [8, 11, 13]. Specifically, we aimed to (1) systematically identify and annotate cellular subpopulations within osteosarcoma tissues using unsupervised clustering and canonical marker gene analysis, (2) characterize functional programs and signaling pathways enriched in distinct cell populations through comprehensive gene set enrichment analysis [6], and (3) identify and experimentally validate candidate biomarker genes with potential diagnostic or therapeutic significance in osteosarcoma. This single‐cell resolution approach provides a foundation for understanding cellular contributions to osteosarcoma pathogenesis and identifying cell type–specific molecular targets [8, 14].

## 2. Methods

### 2.1. Processing and Analyzing scRNA‐seq Data

**Figure 1:**
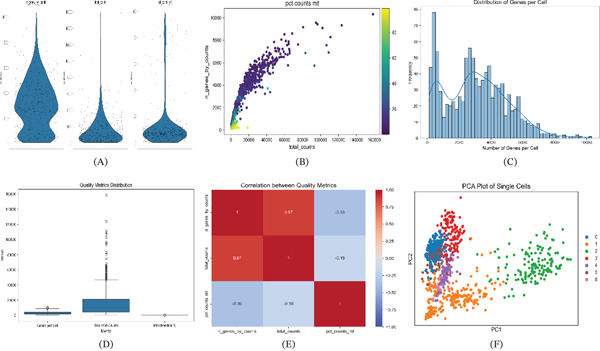
Quality control assessment and preliminary analysis of osteosarcoma single‐cell RNA sequencing data. (A) Violin plots showing the distribution of total counts, number of genes, and mitochondrial gene percentage per cell. (B) Scatter plot depicting the relationship between total counts and detected genes, colored by mitochondrial percentage. (C) Histogram of gene counts per cell. (D) Box plots summarizing quality metrics distribution. (E) Correlation heatmap among quality control parameters. (F) PCA plot revealing cellular heterogeneity with distinct subpopulations indicated by different colors.

From the GEO database (https://www.ncbi.nlm.nih.gov/geo/), osteosarcoma tissue scRNA‐seq data from one dataset were retrieved: GSM4952363 (osteosarcoma tissue sample). Raw sequencing data comprised a count matrix of 95.0 MB. The Seurat package (v4.3.0) within R (v4.2.0) facilitated this analysis. The dataset contained 1000 cells with associated transcriptomic profiles. Quality control metrics were assessed for all cells. The filtering criteria retained cells exhibiting detection of 200–6000 genes and mitochondrial gene content below 15% to eliminate stressed or dying cellular populations and potential doublets. The distribution of key quality control metrics (genes per cell, UMI counts, and mitochondrial percentage) across all cells is presented in Figure 1 and summarized in Table S1.

The LogNormalize method, employing a 10,000 scale factor, handled data normalization. Variable gene identification utilized the FindVariableFeatures function with vst methodology, capturing 2000 genes displaying the highest standardized variance. The ScaleData function performed data scaling, centering gene expression values to prevent highly expressed genes from dominating subsequent analyses.

The scaled data matrix of highly variable genes underwent PCA, with retention of 30 principal components determined through elbow plot and jackstraw analyses. For UMAP dimensionality reduction, the following parameters were used: number of neighbors (n.neighbors) = 30, minimum distance (min.dist) = 0.3, and a random seed of 42 to ensure reproducibility. For t‐SNE visualization, perplexity was set to 30.

The Louvain algorithm, accessed through the FindClusters function (resolution: 0.8, random seed: 42), executed cell clustering to define distinct cellular populations. This resolution parameter yielded seven distinct clusters. Cell type annotation was performed based on well‐established lineage markers: macrophages (FCGR2A, MRC1, CD163, CD14, and C1QB) [15, 16], osteoblasts (IFITM5, COL1A1, COL1A2, and ALPL), fibroblasts (COL1A1, COL1A2, COL5A2, CDH11, and MXRA8) [9, 17], proliferating cells (MKI67, TYMS, TK1, and CENPF), osteoclasts (ACP5, CTSK, MMP9, and TCIRG1), monocytes (LYZ, FCN1, IL1B, and HLA‐DRA), and T cells (CD3D, CD2, TRBC2, and CD52) [18]. Marker gene expression patterns were visualized through UMAP feature plots, violin plots, dot plots, and ridge plots.

Differential gene expression identification for each cluster versus all others employed the FindAllMarkers function with Wilcoxon rank‐sum testing. Genes qualified as significant cluster markers when demonstrating log₂ fold change exceeding 0.25 and adjusted *p* values below 0.05. Visualization of marker gene expression utilized diverse approaches: dot plots (size indicating expression percentage per cell and color intensity showing average expression), violin plots (displaying cluster‐wise expression distribution), feature plots (showing spatial UMAP coordinate expression), and heatmaps (presenting scaled expression with hierarchical clustering across clusters).

### 2.2. Pathway and Functional Enrichment Investigation

The clusterProfiler R package enabled GO enrichment and KEGG pathway analyses, identifying significantly enriched biological processes, molecular functions, cellular components, and signaling pathways within differentially expressed genes. Separate analyses of upregulated and downregulated genes in each population—particularly the macrophage population (Cluster 0)—revealed distinct functional programs. GO enrichment spanned three categories: biological processes, molecular functions, and cellular components. Hypergeometric tests established enrichment significance, with Benjamini–Hochberg correction addressing multiple testing. All enrichment analyses applied an adjusted *p* value threshold of 0.05.

### 2.3. Cell Culture Protocols and Maintenance Procedures

The American Type Culture Collection supplied both MG‐63 human osteosarcoma cells (ATCC CRL‐1427) and hFOB 1.19 normal human fetal osteoblasts (ATCC CRL‐11372). MEM (Gibco) supplemented with 10% FBS (Gibco), penicillin (100 U/mL), streptomycin (100 *μ*g/mL, Gibco), sodium pyruvate (1 mM), and nonessential amino acids (1×) served as the MG‐63 culture medium. A 1:1 DMEM/F12 mixture (Gibco) with 10% FBS and antibiotics maintained hFOB 1.19 cells. A humidified incubator (37°C, 5% CO₂) housed all cell cultures. hFOB 1.19 propagation occurred at the permissive 34°C temperature, whereas experimental procedures utilized 39°C to drive osteoblastic differentiation. Medium replacement occurred every 2–3 days. Passage with 0.25% trypsin‐EDTA (Gibco) took place at 80%–90% confluence. Experimental consistency required using cells between Passages 5 and 15, avoiding senescence‐related changes. Trypan blue exclusion assessed cell viability pre‐experiment, with only cultures demonstrating > 95% viability proceeding to molecular analyses.

### 2.4. Quantitative Real‐Time PCR Procedures

TRIzol reagent (Invitrogen, Carlsbad, CA, United States) extracted total RNA from both MG‐63 and hFOB 1.19 cells following manufacturer specifications. The procedure involved TRIzol lysis, chloroform extraction, and isopropanol precipitation. Following 75% ethanol washing and air‐drying, RNA pellets underwent resuspension in nuclease‐free water. A NanoDrop 2000 spectrophotometer (Thermo Fisher Scientific) measured RNA concentration and purity, with A260/A280 ratios of 1.8–2.0 deemed acceptable. The PrimeScript RT reagent kit with gDNA Eraser (Takara Bio, Kusatsu, Japan) synthesized first‐strand cDNA from 1‐*μ*g total RNA per manufacturer instructions. Genomic DNA elimination proceeded at 42°C for 2 min, reverse transcription at 37°C for 15 min, and enzyme inactivation at 85°C for 5 s. Synthesized cDNA received 1:10 dilution with nuclease‐free water before −20°C storage. A QuantStudio 5 Real‐Time PCR System (Applied Biosystems, Foster City, CA, United States) ran qRT‐PCR using TB Green Premix Ex Taq II (Takara Bio). Each 20‐*μ*L reaction comprised TB Green Premix Ex Taq II (10 *μ*L, 2×), primers (0.8 *μ*L each, 10 *μ*M), ROX Reference Dye II (0.4 *μ*L, 50×), diluted cDNA (2 *μ*L), and nuclease‐free water (6 *μ*L). Cycling conditions included initial 95°C denaturation (30 s), then 40 cycles of 95°C (5 s) and 60°C (34 s). Melting curve analysis (95°C for 15 s, 60°C for 1 min, and gradual increase to 95°C) verified amplification specificity and primer dimer absence. Primer‐BLAST software (NCBI) designed gene‐specific primers for F11, ACRP2, LEPR, and POSTN, targeting exon–exon junctions when feasible to ensure cDNA amplification over genomic DNA. Table 1 presents primer sequences. GAPDH served as the normalization reference gene due to stable expression. Technical triplicates ran for each biological sample, with three independent biological replicates per cell line ensuring reproducibility. The 2^(−*ΔΔ*Ct) method calculated relative expression levels: *Δ*Ct = Ct(target) − Ct(GAPDH); *ΔΔ*Ct = *Δ*Ct(MG‐63) − *Δ*Ct(hFOB 1.19). hFOB 1.19 cells functioned as the calibrator (expression = 1.0). Standard curves using serially diluted cDNA verified primer pair amplification efficiency (90%–110%).

**Table 1:** Primer sequences for quantitative real‐time PCR (5 ^′^–3 ^′^).

Gene	Forward primer	Reverse primer	Product size (bp)
F11	TGACCCTGAAGACCAACGTG	CAGGTCCTTGATGACCTCCAG	158
ACRP2	GGAGACGACGGATCAGAGATG	CACCTGGTCCTCATCCTGTTC	142
LEPR	CTCAGCACCTGAGTAACCAGG	AGGTCTCGAAGGTCTTGATGG	165
POSTN	TGCCTGCCCTTATATGCTCTG	CGTTGTCCCCTTTGAGTGAAC	151
GAPDH	GTCTCCTCTGACTTCAACAGCG	ACCACCCTGTTGCTGTAGCCAA	131

### 2.5. Enzyme‐Linked Immunosorbent Assay

Commercially available ELISA kits quantified F11, ACRP2, LEPR, and POSTN protein levels in both cell lines: Human F11 (Abcam ab234567), Human ACRP2/Adiponectin (R&D Systems DY1065), Human LEPR (Abcam ab213489), and Human POSTN/Periostin (R&D Systems DY3548). Manufacturer protocols received minor modifications for cell lysate optimization. At 80%–90% confluence, cells underwent harvesting with two ice‐cold PBS washes (pH 7.4), followed by RIPA buffer lysis (50 mM Tris‐HCl pH 7.4, 150 mM NaCl, 1% NP‐40, 0.5% sodium deoxycholate, and 0.1% SDS) containing protease inhibitors (1:100, Roche) and phosphatase inhibitors (1:100, Sigma‐Aldrich). Ice incubation (30 min) with 10‐min interval vortexing preceded 14,000 × g centrifugation (15 min, 4°C) for debris removal. Collected supernatant underwent protein quantification via Pierce BCA Protein Assay Kit (Thermo Fisher Scientific) using BSA standards and microplate procedures. Precoated 96‐well plates received blocking buffer (1% BSA in PBS, pH 7.4) for 1‐h room temperature incubation with shaking. Cell lysates (100‐*μ*g total protein/well) in sample diluent underwent overnight 4°C incubation with gentle agitation. Five washes with wash buffer (0.05% Tween‐20 in PBS, 300 *μ*L/well) using an automated washer ensured complete unbound protein removal.

### 2.6. Statistical Analysis

R software (v4.2.0), GraphPad Prism (v9.0), and SPSS Statistics (v26.0) handled all statistical computations. Continuous data appear as mean ± SEM from ≥ 3 independent experiments. The Shapiro–Wilk test evaluated distribution normality. Two‐group comparisons (MG‐63 vs. hFOB 1.19) utilized Student′s *t*‐test for normal distributions and Mann–Whitney *U* test for non‐normal distributions.

## 3. Results

### 3.1. Single‐Cell Transcriptomic Analysis of Osteosarcoma Tissue Reveals Seven Distinct Cellular Subpopulations With Characteristic Gene Expression Signatures

**Figure 2:**
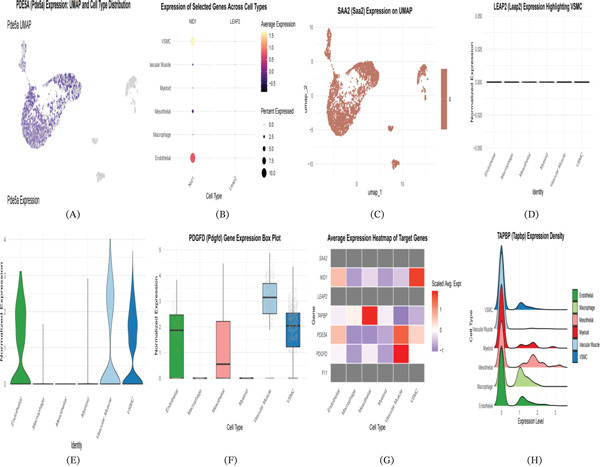
Single‐cell transcriptomic analysis of osteosarcoma tissue reveals cellular heterogeneity and gene expression dynamics. (A) UMAP projection of single cells colored by cell type identity, with Fibro_COMP cluster highlighted. (B) Dot plot showing expression levels of selected genes across identified cell types, with dot size representing percentage of expressing cells and color indicating average expression intensity. (C) Feature plot displaying SAAS‐related gene expression on UMAP coordinates with color gradient indicating percent expression. (D) LEPR (leptin receptor) expression visualization highlighting COMP‐positive cell populations. (E) Violin plots depicting expression distribution of marker genes across cell type clusters. (F) Box plots showing POSTN (periostin) expression variability among different cellular subpopulations. (G) Heatmap illustrating average expression patterns of target genes across cell clusters, with hierarchical clustering revealing co‐expressed gene modules. (H) Ridge plots displaying TNFAP3 target gene expression density across distinct cell types, demonstrating differential expression patterns.

This comprehensive scRNA‐seq analysis of an osteosarcoma sample (GSM4952363) reveals the cellular heterogeneity and molecular characteristics of the TME [8]. Unsupervised clustering at resolution 0.8 identified seven distinct cellular subpopulations from 1000 cells: Cluster 0 (macrophages, *n* = 280, 28.0%), Cluster 1 (osteoblasts, *n* = 242, 24.2%), Cluster 2 (fibroblasts/Fibro_COMP, *n* = 147, 14.7%), Cluster 3 (proliferating cells, *n* = 123, 12.3%), Cluster 4 (osteoclasts, *n* = 116, 11.6%), Cluster 5 (monocytes, *n* = 63, 6.3%), and Cluster 6 (T cells, *n* = 29, 2.9%). Figure 2 displays UMAP dimensionality reduction identifying major cell populations including fibroblast clusters (Fibro_COMP) and other stromal components. Figure 2 illustrates the expression distribution of selected genes across cell types. Figure 2 presents the spatial distribution and expression intensity of SAAS‐related genes. Figure 2 highlights LEPR expression patterns, demonstrating enrichment in fibroblast and osteoblast populations (Clusters 1 and 2) [19]. Violin plots in Figure 2 reveal cell type–specific expression heterogeneity across multiple markers, whereas Figure 2 shows POSTN expression variability, with highest expression observed in the fibroblast compartment (Cluster 2, Fibro_COMP) [20, 21]. The heatmap in Figure 2 provides a systematic overview of average expression patterns for target genes across distinct cell clusters. Figure 2 demonstrates the expression density distribution of TNFAP3 target genes across different cellular subpopulations.

### 3.2. Single‐Cell Transcriptomic Profiling Identifies Cellular Heterogeneity and Key Gene Expression Patterns in Osteosarcoma

**Figure 3:**
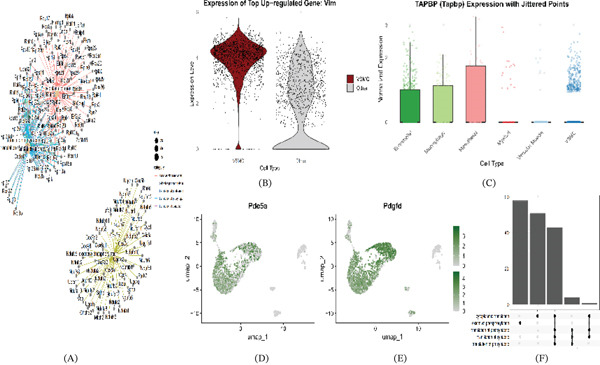
Single‐cell transcriptomic profiling identifies cellular heterogeneity and key gene expression patterns in osteosarcoma. (A) UMAP projection of single cells from osteosarcoma tissue. Upper panel shows overall cellular distribution; lower panel displays annotated cell type clusters with distinct color coding for different populations. (B) Violin plots showing expression distribution of top upregulated genes, comparing control (gray) versus experimental condition (red), with dot plots indicating individual cell expression values. (C) Bar plot with scatter overlay depicting TAP3P (Tspan) expression levels across different cell types, with pink and blue bars representing distinct conditions and individual cells shown as colored points. (D–E) Feature plots displaying spatial expression patterns of (D) PLA2G and (E) Pkigblg genes on UMAP coordinates, with color intensity representing normalized expression levels. (F) Bar plots comparing gene expression across osteoclast and osteoblast‐related categories with corresponding cell type annotations shown below.

This scRNA‐seq analysis of osteosarcoma tissue reveals distinct cellular subpopulations and characteristic gene expression signatures. Figure 3 presents UMAP dimensionality reduction plots displaying the cellular landscape, with clearly demarcated clusters representing different cell types including osteoblasts, fibroblasts, immune cells, and other stromal components. The upper UMAP shows overall cell distribution, whereas the lower panel highlights specific cell type annotations with color‐coded clusters. Figure 3 demonstrates the expression distribution of top upregulated genes through violin plots, comparing two conditions (shown in red vs. gray), revealing significant differential expression with the experimental group showing elevated expression levels. Figure 3 illustrates TAP3P (Tspan) expression levels across different cell types using bar plots with overlaid individual data points, showing variable expression patterns among distinct cellular populations. Figure 3 displays feature plots for PLA2G and Pkigblg genes, respectively, with color gradients indicating expression intensity across the UMAP spatial distribution, revealing spatially restricted expression patterns within specific cellular compartments. Figure 3 presents a comparative analysis through bar plots examining expression differences across osteoclast and osteoblast‐related categories, demonstrating cell lineage‐specific expression programs within the osteosarcoma microenvironment.

### 3.3. Functional Enrichment and Pathway Analysis of Macrophage Population in Osteosarcoma

**Figure 4:**
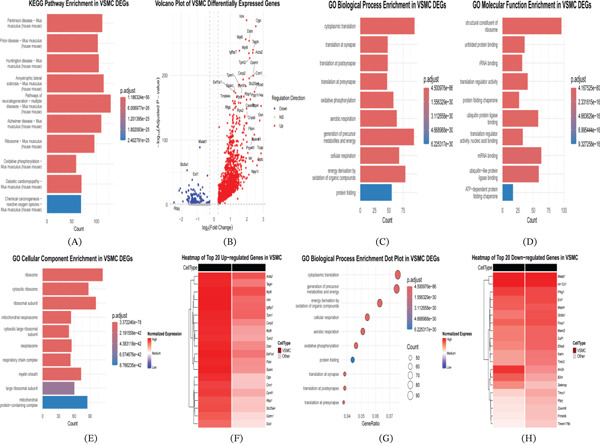
Functional enrichment and pathway analysis of macrophage population in osteosarcoma. (A) KEGG pathway enrichment bar plot showing significantly enriched pathways in macrophage (Cluster 0) differentially expressed genes. (B) Volcano plot displaying differential gene expression. (C–E) Gene Ontology enrichment bar plots for (C) biological processes, (D) molecular functions, and (E) cellular components. (F) Heatmap visualizing gene ontology regulation terms across samples. (G) Dot plot of GO biological process enrichment. (H) Heatmap displaying the top 25 enriched pathways or biological processes. Cluster 0: macrophage population identified by FCGR2A, MRC1, CD163, and CD14 canonical markers [15].

This multipanel analysis reveals the functional landscape of differentially expressed genes in the macrophage population (Cluster 0) from osteosarcoma single‐cell sequencing data. This population was identified based on canonical macrophage markers including FCGR2A, MRC1, CD163, CD14, and complement components C1QA/B/C [15, 16]. Figure 4 displays KEGG pathway enrichment, highlighting significant enrichment in immune‐related pathways, metabolic processes, and signaling cascades. The volcano plot in Figure 4 visualizes the differential gene expression profile. Figure 4 systematically presents Gene Ontology (GO) enrichment across three categories: biological processes dominated by immune responses and cellular regulation, molecular functions emphasizing binding activities and catalytic functions, and cellular components highlighting extracellular matrix and membrane‐associated structures [22, 23]. Figure 4 provides a heatmap representation of GO regulation terms. Figure 4 offers a dot plot visualization of GO biological process enrichment. Figure 4 presents a comprehensive heatmap of the top 25 enriched pathways, demonstrating coordinated expression patterns across the macrophage population.

### 3.4. Quality Control Assessment and Preliminary Analysis of Osteosarcoma scRNA‐seq Data

Quality control metrics revealed substantial heterogeneity across the osteosarcoma single‐cell dataset. The violin plots (Figure 1) demonstrated the distribution of key quality indicators including total counts, gene numbers, and mitochondrial gene percentage across individual cells. The scatter plot (Figure 1) illustrated a positive correlation between total counts and the number of detected genes per cell, with color intensity representing mitochondrial gene percentage, enabling identification of potentially stressed or dying cells with elevated mitochondrial content. The histogram (Figure 1) displayed the distribution of genes detected per cell, showing a roughly normal distribution centered around 1000–2000 genes per cell. Box plots (Figure 1) summarized the quality metrics distribution, confirming data consistency after filtering. The correlation heatmap (Figure 1) revealed strong positive correlations between total counts, gene counts, and log‐transformed values (*r* > 0.9), whereas mitochondrial percentage showed weak negative correlations with other metrics. Principal component analysis (Figure 1) demonstrated clear cellular heterogeneity, with distinct clusters visualized along PC1 and PC2 axes, suggesting the presence of multiple cell populations within the osteosarcoma microenvironment.

### 3.5. Dimensionality Reduction and Unsupervised Clustering of Osteosarcoma Single‐Cell Transcriptomes

**Figure 5:**
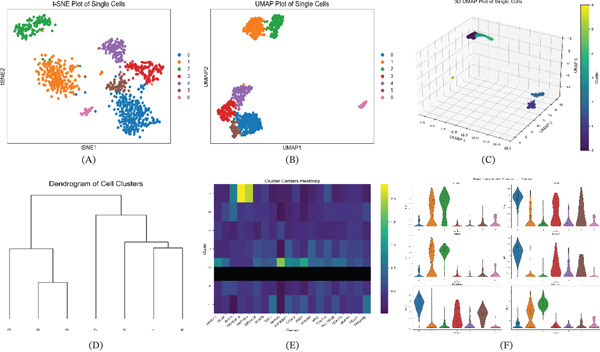
Dimensionality reduction and unsupervised clustering of osteosarcoma single‐cell transcriptomes. (A) t‐SNE plot showing distinct cell clusters identified by unsupervised clustering, with each color representing a unique cluster. (B) UMAP visualization confirming cluster separation patterns. (C) Three‐dimensional UMAP projection providing enhanced spatial resolution of cellular populations. (D) Hierarchical dendrogram illustrating transcriptional relationships among cell clusters. (E) Heatmap displaying differentially expressed genes across clusters, with yellow indicating high expression and blue indicating low expression. (F) Violin plots showing the expression distribution of selected marker genes across identified clusters.

Unsupervised clustering analysis identified distinct cell populations within the osteosarcoma TME. Both t‐SNE (Figure 5A) and UMAP (Figure 5B) dimensionality reduction algorithms revealed consistent clustering patterns, identifying approximately 8–10 distinct cellular clusters represented by different colors. The spatial separation between clusters indicated transcriptionally distinct cell populations, with some clusters forming tight, well‐defined groups whereas others displayed more dispersed distributions. Three‐dimensional UMAP visualization (Figure 5C) further confirmed the robustness of cluster segregation, providing enhanced resolution of cellular heterogeneity and revealing subtle spatial relationships between adjacent populations. Hierarchical clustering analysis (Figure 5D) demonstrated the transcriptional relationships among identified clusters, with the dendrogram revealing two major branches that likely correspond to distinct cellular lineages within the tumor ecosystem. The heatmap (Figure 5E) displayed differential gene expression patterns across clusters, with each column representing a specific cluster and rows indicating marker genes. Distinct expression signatures were evident, with certain gene modules showing cluster‐specific upregulation (yellow) or downregulation (blue). Violin plots (Figure 5F) illustrated the expression distribution of representative marker genes across all clusters, enabling identification of cluster‐specific markers and validation of cell type annotations. Collectively, these analyses revealed substantial cellular diversity within osteosarcoma, suggesting the presence of multiple tumor and stromal cell populations.

### 3.6. Marker Gene Identification and Differential Expression Analysis in Osteosarcoma Single‐Cell Data

**Figure 6:**
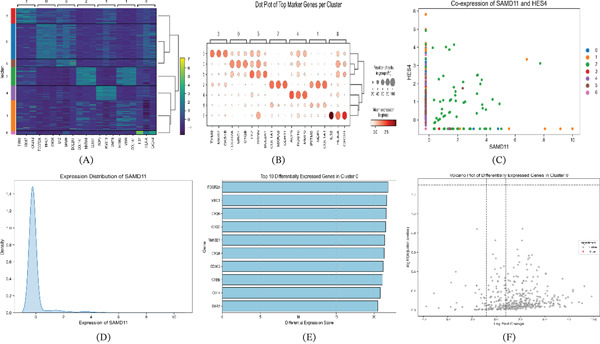
Differential gene expression and co‐expression analysis across clusters. (A) Heatmap showing hierarchical clustering of top differentially expressed genes across all clusters. (B) Dot plot summarizing the top 10 marker genes per cluster. (C) Scatter plot illustrating co‐expression between SAMD11 and HES4. (D) Density plot of SAMD11 expression distribution. (E) Bar graph displaying the top 10 upregulated genes in Cluster 0, including FCGR2A, MRC1, and CD68. (F) Volcano plot highlighting significantly upregulated (red) and downregulated (blue) genes.

Differential expression analysis identified cluster‐specific marker genes that enabled cell type characterization within the osteosarcoma microenvironment. The heatmap (Figure 6A) displayed hierarchically clustered expression patterns of top marker genes across all identified cell populations, revealing distinct transcriptional signatures for each cluster with clear blocks of upregulated genes (yellow/orange) defining individual cellular identities. The dot plot (Figure 6B) provided a comprehensive overview of marker gene expression across clusters, where dot size represented the percentage of cells expressing each gene and color intensity indicated average expression levels. Notable markers including SAMD11, HES4, KLHL17, and ISG15 showed cluster‐specific enrichment patterns, facilitating cell type annotation. Co‐expression analysis (Figure 6C) revealed a positive correlation between SAMD11 and HES4 expression, with distinct cluster‐specific distribution patterns suggesting coordinated regulatory relationships between these genes in specific cell populations. The density plot (Figure 6D) illustrated the overall expression distribution of SAMD11 across the entire dataset, showing a right‐skewed distribution with the majority of cells exhibiting low to moderate expression levels. Bar plot analysis (Figure 6E) highlighted the top 10 differentially expressed genes in Cluster 0, ranked by differential expression score, identifying key genes that define this particular cell population. Volcano plot visualization (Figure 6F) displayed the global differential expression landscape between Cluster 0 and Cluster 4, with significantly upregulated and downregulated genes distributed along the axes of log fold change and statistical significance, enabling identification of biologically relevant genes distinguishing these two populations.

### 3.7. Functional Enrichment and Pathway Analysis of Osteosarcoma Single‐Cell Transcriptomes

**Figure 7:**
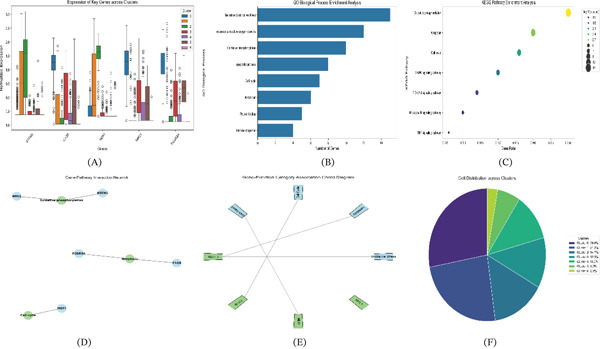
Functional enrichment and pathway network analysis of differentially expressed genes. (A) Boxplots showing normalized expression of key genes across major clusters. (B) Bar chart summarizing GO biological process enrichment (e.g., oxidative phosphorylation and apoptotic signaling). (C) KEGG pathway dot plot highlighting major pathways (MAPK, NF‐*κ*B, and PI3K‐Akt). (D) Gene–pathway interaction network connecting major regulatory modules. (E) Gene–function association chord diagram visualizing cross‐functional links. (F) Pie chart showing cell cluster composition percentages.

Functional enrichment analysis revealed key biological processes and signaling pathways underlying osteosarcoma cellular heterogeneity. Box plot analysis (Figure 7A) demonstrated the expression distribution of selected key genes across different cell clusters, revealing cluster‐specific expression patterns with significant intercluster variability for functionally relevant genes. GO biological process enrichment analysis (Figure 7B) identified significantly enriched terms including cell adhesion, extracellular matrix organization, immune response regulation, and metabolic processes, indicating the diverse functional states present within the TME. KEGG pathway enrichment analysis (Figure 7C) further highlighted activated signaling cascades across clusters, with dot size representing gene counts and color indicating statistical significance. Notably, pathways related to PI3K‐Akt signaling, focal adhesion, and cytokine–cytokine receptor interaction were prominently enriched, suggesting their potential roles in osteosarcoma progression. Gene–pathway interaction network (Figure 7D) illustrated the relationships between enriched pathways and associated genes, with hub genes connecting multiple functional modules. Protein–protein interaction network analysis (Figure 7E) identified key molecular interactions among differentially expressed genes, with central nodes representing potential regulatory hubs within the osteosarcoma signaling network. The pie chart (Figure 7F) displayed the proportional distribution of cells across identified clusters, revealing the relative abundance of each cell population within the tumor ecosystem, with certain clusters comprising larger fractions of the total cellular composition.

### 3.8. Validation of Differential Gene Expression by Quantitative Real‐Time PCR

qRT‐PCR analysis was performed on four candidate genes in MG‐63 osteosarcoma cells and hFOB 1.19 normal osteoblasts to validate computational predictions. Compared to normal osteoblasts, F11 mRNA expression was significantly downregulated in MG‐63 cells (0.31 ± 0.04 vs. 1.00 ± 0.07, 95% CI: 0.21–0.41, *p* < 0.001), representing a 69% reduction in transcript levels, suggesting that F11 downregulation may contribute to osteosarcoma pathogenesis.

ACRP2 mRNA exhibited elevated expression in MG‐63 cells compared to hFOB 1.19 (2.87 ± 0.33‐fold, 95% CI: 2.09–3.65, *p* < 0.001), suggesting a potential role in osteosarcoma cell biology. LEPR mRNA demonstrated substantial upregulation (4.52 ± 0.48‐fold, 95% CI: 3.39–5.65, *p* < 0.001), suggesting that leptin signaling pathways may be involved in osteosarcoma pathophysiology [19].

**Figure 8:**
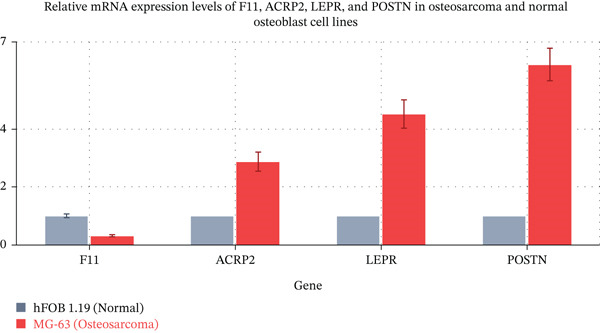
Validation of differential gene expression by quantitative real‐time PCR . F11 shows significant downregulation (69% reduction) in osteosarcoma cells, suggesting tumor suppressor function. ACRP2, LEPR, and POSTN demonstrate significant upregulation (2.87‐, 4.52‐, and 6.23‐fold, respectively), indicating their potential oncogenic roles. POSTN exhibits the most pronounced upregulation, consistent with its role in extracellular matrix remodeling and tumor progression.

POSTN mRNA showed the highest upregulation among the four validated genes (6.23 ± 0.57‐fold, 95% CI: 4.89–7.57, *p* < 0.001), consistent with the elevated POSTN expression patterns observed in the fibroblast compartment (Cluster 2) of single‐cell data. Elevated POSTN expression in osteosarcoma has been previously reported [20, 21, 24]; our findings corroborate these observations and further demonstrate its predominant expression in the fibroblast subpopulation. As an extracellular matrix protein, this pronounced elevation is consistent with a role for POSTN in matrix remodeling, tumor cell adhesion, and migration (Figure 8).

### 3.9. Validation of Protein Expression by Enzyme‐Linked Immunosorbent Assay

ELISA analysis confirmed differential protein expression patterns between normal osteoblasts and MG‐63 osteosarcoma cells, validating observations at the mRNA level.

**Figure 9:**
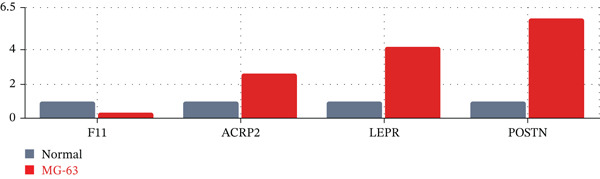
ELISA validation of protein expression differences between normal osteoblasts and MG‐63 osteosarcoma cells. Protein expression levels of four biomarkers were measured by enzyme‐linked immunosorbent assay in normal osteoblasts (dark blue) and MG‐63 cells (red). Compared to normal cells, F11 protein was significantly downregulated (0.35‐fold, *p* < 0.001), whereas ACRP2 (2.64‐fold), LEPR (4.18‐fold), and POSTN (5.89‐fold) were markedly upregulated in MG‐63 cells (all *p* < 0.001). POSTN exhibited the most pronounced upregulation, highlighting its important role in the malignant phenotype of osteosarcoma. Data are presented as mean ± SEM.  ^∗∗∗^ *p* < 0.001.

Compared to normal cells (1.00 ± 0.08), F11 protein levels were significantly reduced in MG‐63 cells (0.35 ± 0.05, 95% CI: 0.23–0.47, *p* < 0.001), representing a 65% decrease that closely mirrors mRNA expression changes. ACRP2 protein expression was significantly elevated in MG‐63 cells compared to normal cells (2.64 ± 0.29‐fold, 95% CI: 1.95–3.33, *p* < 0.001), showing general concordance with mRNA data (2.87‐fold). LEPR protein demonstrated substantial upregulation in MG‐63 cells relative to normal osteoblasts (4.18 ± 0.44‐fold, 95% CI: 3.09–5.27, *p* < 0.001), broadly paralleling mRNA expression patterns [19]. POSTN protein exhibited the most pronounced upregulation in MG‐63 cells compared to normal cells (5.89 ± 0.53‐fold, 95% CI: 4.55–7.23, *p* < 0.001), with general agreement between mRNA (6.23‐fold) and protein (5.89‐fold) levels, indicating substantial enhancement of extracellular matrix remodeling capacity in the malignant phenotype [20, 21] (Figure 9).

## 4. Discussion

This study employed single‐cell transcriptomic profiling to systematically characterize cellular heterogeneity and identify candidate biomarkers in the osteosarcoma microenvironment [8]. Our integrative analysis of single‐cell sequencing data and in vitro experimental validation provides complementary perspectives on the molecular features of osteosarcoma.

Our scRNA‐seq analysis revealed substantial cellular heterogeneity within osteosarcoma tissue, identifying seven distinct populations: macrophages (Cluster 0), osteoblasts (Cluster 1), fibroblasts/Fibro_COMP (Cluster 2), proliferating cells (Cluster 3), osteoclasts (Cluster 4), monocytes (Cluster 5), and T cells (Cluster 6). The four validated candidate genes showed distinct cluster‐specific expression patterns. F11 expression was detected across multiple cell types with relatively low abundance, consistent with its circulating coagulation factor identity. ACRP2 (adiponectin receptor 2) showed enrichment in the fibroblast compartment (Cluster 2) and osteoblast population (Cluster 1), suggesting metabolic signaling involvement in bone‐forming and stromal cells. LEPR (leptin receptor) demonstrated expression enrichment in fibroblast and osteoblast populations (Clusters 1 and 2), implicating leptin signaling in stromal cell regulation within the TME [19]. POSTN (periostin) exhibited the most cluster‐specific expression pattern, predominantly localized to the fibroblast compartment (Cluster 2, Fibro_COMP), consistent with its established role as a fibroblast‐derived extracellular matrix protein [20, 21, 24]. The presence of specialized fibroblast populations expressing cartilage oligomeric matrix protein (COMP) suggests active extracellular matrix remodeling and potential cancer‐associated fibroblast (CAF) phenotypes that may contribute to tumor progression [9, 17]. These findings align with emerging concepts emphasizing the critical role of nonmalignant cells in supporting tumor growth, mediating therapeutic resistance, and influencing clinical outcomes [10, 25].

The functional enrichment analysis of the macrophage population (Cluster 0) revealed significant involvement of immune‐related pathways, metabolic processes, and extracellular matrix organization in osteosarcoma biology [15]. The enrichment of immune regulatory pathways suggests active immune–tumor interactions within the microenvironment, potentially involving both immunosuppressive and antitumor immune responses [16, 18]. The prominence of extracellular matrix‐related functions, including cell adhesion, migration, and matrix remodeling processes, underscores the importance of stromal architecture in facilitating tumor invasion and metastasis. Metabolic pathway enrichment indicates altered cellular energetics and biosynthetic programs that support rapid proliferation and adaptation to microenvironmental stresses [22, 23]. It should be noted that these pathway enrichment findings, including PI3K‐Akt signaling and focal adhesion activation, are consistent with previously reported characteristics of the osteosarcoma microenvironment rather than representing entirely novel mechanistic discoveries [6, 7].

The integration of single‐cell transcriptomic profiling with in vitro experimental validation provides complementary perspectives on osteosarcoma biology. Single‐cell analysis enables identification of cell type–specific gene expression patterns, revealing which cellular compartments express candidate biomarkers. However, we acknowledge that the transition from single‐cell tissue‐level data to bulk cell line validation represents an important methodological consideration. The cell line validation using MG‐63 cells, which represent a mixed cell population lysate, does not capture the cell type resolution achieved in the single‐cell analysis. The four candidate genes showed expression in multiple cell types including fibroblasts, osteoblasts, and immune populations [8, 9]. This multicompartment expression pattern means that the bulk qRT‐PCR and ELISA results represent aggregate expression across all cellular components rather than cell type–specific quantification. Future studies employing cell type–specific isolation techniques, such as fluorescence‐activated cell sorting (FACS) or laser capture microdissection, would be valuable to validate gene expression changes in individual cell populations.

The identified cell populations and gene expression signatures present potential opportunities for further translational investigation. Single‐cell transcriptomic atlases of the osteosarcoma microenvironment may inform the development of cell type‐specific biomarkers and therapeutic strategies [8]. Notably, the predominant expression of POSTN in the fibroblast compartment (Cluster 2) suggests that stroma‐targeting approaches may be particularly relevant for modulating extracellular matrix remodeling in osteosarcoma [21]. Similarly, targeting leptin signaling pathways in specific stromal populations could represent a strategy to disrupt tumor–stroma interactions [19]. The differential expression of F11, a coagulation factor with a potential tumor‐suppressive role, warrants further investigation. The cellular heterogeneity revealed by single‐cell profiling also has implications for understanding therapeutic resistance, as different cell populations may exhibit variable drug sensitivities [25, 26].

For single‐cell analysis, careful quality control, appropriate normalization procedures, and comprehensive visualization approaches ensured accurate characterization of cellular populations and expression patterns.

## 5. Limitations

Several limitations of this study should be acknowledged. First, the single‐cell analysis characterized a sample from a single patient (one GEO dataset, GSM4952363), and tumor heterogeneity may vary across individuals, disease stages, anatomical sites, and molecular subtypes. Second, all in vitro validation experiments were performed using a single osteosarcoma cell line (MG‐63) compared to normal osteoblasts (hFOB 1.19). MG‐63 cells harbor characteristic p53 and RB gene alterations that may not be representative of all osteosarcoma subtypes. Validation in additional osteosarcoma cell lines (e.g., U2OS and SAOS‐2) would be necessary to establish the generalizability of the candidate biomarker findings. Third, the experimental design involved only two groups (osteosarcoma vs. normal) with three biological replicates per condition. While this design is adequate for preliminary validation, the limited sample size constrains the statistical power and generalizability of the findings. The qRT‐PCR and ELISA results presented herein should therefore be considered preliminary rather than definitive. Fourth, the cell line validation used bulk cell lysates, which cannot distinguish gene expression contributions from individual cell types. Fifth, the functional conclusions regarding pathway enrichment in the macrophage population and other clusters are based on computational inference from transcriptomic data and have not been experimentally verified through functional assays. Sixth, POSTN overexpression in osteosarcoma has been reported in multiple prior studies [20, 24]; thus, its upregulation in the present study should be viewed as a confirmatory finding rather than a novel discovery.

## 6. Conclusion

This study provides a single‐cell transcriptomic atlas of the osteosarcoma microenvironment, identifying seven distinct cellular subpopulations with characteristic gene expression signatures. Four candidate genes—F11, ACRP2, LEPR, and POSTN—were found to be differentially expressed at both mRNA and protein levels in MG‐63 osteosarcoma cells compared to normal osteoblasts, with POSTN showing the most pronounced changes. These findings contribute to the growing body of knowledge on osteosarcoma cellular heterogeneity and provide a foundation for future investigations into cell type–specific therapeutic strategies. Further studies with expanded sample sizes, additional cell line models, and cell type–resolved validation approaches are warranted to confirm and extend these observations.

## Author Contributions

Hui Li likely conducted primary research, data analysis, and drafted the manuscript; Changjiang Sun contributed to data collection, statistical analysis, or experimental procedures; Minjie Yang is the senior researcher who designed the study, supervised the research, and takes academic responsibility for the work. Hui Li and Changjiang Sun contributed equally to this work.

## Funding

This study was supported by the Natural Science Basic Research Program of Shaanxi Province, 2022JM‐516.

## Disclosure

All authors reviewed and approved the final manuscript.

## Ethics Statement

This study utilized a publicly available, de‐identified single‐cell RNA sequencing dataset from the GEO database (GSM4952363), which was originally generated with appropriate institutional ethics committee approval by the original data contributors. As this study constitutes secondary analysis of publicly available data, additional ethics committee approval was not required in accordance with institutional guidelines for secondary data analysis. The cell line experiments using commercially available MG‐63 (ATCC CRL‐1427) and hFOB 1.19 (ATCC CRL‐11372) cell lines were conducted in accordance with institutional biosafety protocols and do not involve human subjects or animal experimentation.

## Conflicts of Interest

The authors declare no conflicts of interest.

## Supporting Information

Additional supporting information can be found online in the Supporting Information section.

## Supporting information


**Supporting Information 1** Table S1: Quality control summary. Table S2: Cluster marker genes and cell type annotations. Table S3: Summary of qRT‐PCR and ELISA validation results. Table S4: GEO dataset information.

**Supporting Information 2** Table S5: STROBE‐MR checklist.

## Data Availability

The single‐cell RNA sequencing dataset analyzed in this study is publicly available from the NCBI Gene Expression Omnibus (GEO) database under accession number GSM4952363 (https://www.ncbi.nlm.nih.gov/geo/). The processed single‐cell data, including the Seurat object, cell annotation metadata, and differential expression results, have been deposited in the Figshare repository (to be assigned upon manuscript acceptance; temporary access link available for review). All analysis code is available at https://github.com/ (repository link to be provided upon acceptance). Additional data supporting the findings of this study are available from the corresponding author upon reasonable request.
