# Supplementary material for: Single‐Cell Transcriptomic Profiling Reveals Cellular Heterogeneity and Identifies Novel Therapeutic Targets in Osteosarcoma
Source: Int J Genomics. 2026 Jul 18;2026:4040246. doi: 10.1155/ijog/4040246 (PMC13379946; doi:10.1155/ijog/4040246)
Supplement: Supplementary file 1 — Supporting Information 1 Table S1: Quality control summary. Table S2: Cluster marker genes and cell type annotations. Table S3: Summary of qRT‐PCR and ELISA validation results. Table S4: GEO dataset information. [file IJOG-2026-4040246-s001.docx]

**Supplementary Materials**

**Supplementary Tables for:** "Single-Cell Transcriptomic Profiling Reveals Cellular Heterogeneity and Identifies Novel Therapeutic Targets in Osteosarcoma"

**Supplementary Table S1. Quality Control Summary**

| **Parameter** | **Value** |
| --- | --- |
| Total cells initially detected | 1,000 |
| Cells passing QC (200 ≤ genes ≤ 6,000; MT < 15%) | 795 (79.5%) |
| Cells excluded: < 200 genes | 16 (1.6%) |
| Cells excluded: > 6,000 genes | 67 (6.7%) |
| Cells excluded: MT > 15% | 142 (14.2%) |
| Mean genes per cell (post-QC) | 3039 |
| Mean UMIs per cell (post-QC) | 13497 |

QC filtering criteria: cells with 200-6,000 detected genes and < 15% mitochondrial gene content were retained for downstream analysis. Note that some cells failed multiple criteria.

**Supplementary Table S2. Cluster Marker Genes and Cell Type Annotations**

| **Cluster** | **Cell Type** | **Cell Count (%)** | **Canonical Markers** | **Top 5 DEGs** |
| --- | --- | --- | --- | --- |
| 0 | Macrophages | 280 (28.0%) | FCGR2A, MRC1, CD163, CD14, C1QA, C1QB, C1QC | FCGR2A, MRC1, C1QB, C1QC, RNASE1 |
| 1 | Osteoblasts | 242 (24.2%) | IFITM5, COL1A1, COL1A2, ALPL, MDFI, CDK4 | SRGN, CTSL, HLA-DRB1, CD74, LYZ |
| 2 | Fibroblasts (Fibro_COMP) | 147 (14.7%) | COL1A1, COL1A2, COL5A2, CDH11, MXRA8, PCOLCE, TWIST1 | COL1A1, MXRA8, CDH11, COL1A2, COL5A2 |
| 3 | Proliferating Cells | 123 (12.3%) | MKI67, TYMS, TK1, CENPF, SMC4, MAD2L1, NUSAP1 | TYMS, MKI67, CKS1B, SMC4, TK1 |
| 4 | Osteoclasts | 116 (11.6%) | ACP5, CTSK, MMP9, TCIRG1, SIGLEC15, ATP6V0D2 | ACP5, RGS10, MMP9, TCIRG1, CTSK |
| 5 | Monocytes | 63 (6.3%) | LYZ, FCN1, IL1B, HLA-DRA, SRGN, BCL2A1, EREG, AREG | LYZ, SRGN, BCL2A1, EREG, PLAUR |
| 6 | T Cells | 29 (2.9%) | CD3D, CD2, TRBC2, CD52, IL32, CXCR4, LTB | IL32, HLA-A, CXCR4, CD2, CD3D |

DEGs: differentially expressed genes (|log₂FC| > 0.25, adjusted P < 0.05, Wilcoxon rank-sum test). Canonical markers listed are well-established lineage markers from the literature used for cell type annotation.

**Supplementary Table S3. Summary of qRT-PCR and ELISA Validation Results**

| **Gene** | **qRT-PCR Fold Change** | **qRT-PCR 95% CI** | **ELISA Fold Change** | **ELISA 95% CI** | **Direction** | **P-value** |
| --- | --- | --- | --- | --- | --- | --- |
| F11 | 0.31 ± 0.04 | 0.21–0.41 | 0.35 ± 0.05 | 0.23–0.47 | Downregulated | < 0.001 |
| ACRP2 | 2.87 ± 0.33 | 2.09–3.65 | 2.64 ± 0.29 | 1.95–3.33 | Upregulated | < 0.001 |
| LEPR | 4.52 ± 0.48 | 3.39–5.65 | 4.18 ± 0.44 | 3.09–5.27 | Upregulated | < 0.001 |
| POSTN | 6.23 ± 0.57 | 4.89–7.57 | 5.89 ± 0.53 | 4.55–7.23 | Upregulated | < 0.001 |

qRT-PCR values represent mRNA expression levels (fold change relative to hFOB 1.19 calibrator, GAPDH-normalized, 2^(-ΔΔCt) method). ELISA values represent protein expression levels (fold change relative to hFOB 1.19 controls, normalized to total protein). Data are mean ± SEM from three independent biological replicates with three technical replicates each. CI: confidence interval.

**Supplementary Table S4. GEO Dataset Information**

| **Accession** | **Sample Type** | **Platform** | **Cells Analyzed** |
| --- | --- | --- | --- |
| GSM4952363 | Osteosarcoma tissue | 10x Genomics | 1,000 |
