# Supplementary material for: Single‐Cell Transcriptomic Profiling Reveals Cellular Heterogeneity and Identifies Novel Therapeutic Targets in Osteosarcoma
Source: Int J Genomics. 2026 Jul 18;2026:4040246. doi: 10.1155/ijog/4040246 (PMC13379946; doi:10.1155/ijog/4040246)
Supplement: Supplementary file 2 — Supporting Information 2 Table S5: STROBE‐MR checklist. [file IJOG-2026-4040246-s002.docx]

**STROBE-MR checklist of recommended items to address in reports of Mendelian randomization studies**^1^ ^2^

| **Item No.** | **Section** | **Checklist item** | **Page No.** | **Relevant text from manuscript** |
| --- | --- | --- | --- | --- |
| 1 | **TITLE and ABSTRACT** | Indicate Mendelian randomization (MR) as the study’s design in the title and/or the abstract if that is a main purpose of the study | 1-2 | Title/abstract do not identify MR as the study design. The title is “Single-Cell Transcriptomic Profiling…”; the abstract describes scRNA-seq analysis and qRT-PCR/ELISA validation. |
|  | **INTRODUCTION** |  |  |  |
| 2 | **Background** | Explain the scientific background and rationale for the reported study. What is the exposure? Is a potential causal relationship between exposure and outcome plausible? Justify why MR is a helpful method to address the study question | 2-3 | Background discusses osteosarcoma, then states that “Mendelian randomization (MR) has emerged as a powerful genetic epidemiological approach…” and explains why MR could help address causal questions. |
| 3 | **Objectives** | State specific objectives clearly, including pre-specified causal hypotheses (if any). State that MR is a method that, under specific assumptions, intends to estimate causal effects | 3 | Objectives state that the study aimed to “conduct comprehensive MR analysis…; perform single-cell RNA sequencing…; and integrate genetic and cellular insights…”. |
|  | **METHODS** |  |  |  |
| 4 | **Study design and data sources** | Present key elements of the study design early in the article. Consider including a table listing sources of data for all phases of the study. For each data source contributing to the analysis, describe the following: | 3-8 | Methods present data retrieval, scRNA-seq processing, enrichment analysis, cell experiments, qRT-PCR, ELISA, and statistics; however, a dedicated MR study design/data-source description is not reported. |
|  | a) | Setting: Describe the study design and the underlying population, if possible. Describe the setting, locations, and relevant dates, including periods of recruitment, exposure, follow-up, and data collection, when available. | 4 | Setting/data source reported only for scRNA-seq: “From the GEO database, osteosarcoma tissue single-cell RNA sequencing data (GSM8776323 and GSM4952363) underwent retrieval…”. No recruitment dates/locations are given. |
|  | b) | Participants: Give the eligibility criteria, and the sources and methods of selection of participants. Report the sample size, and whether any power or sample size calculations were carried out prior to the main analysis | 4 | Cell-level inclusion/QC criteria are reported (>200 and <6000 genes/cell; mitochondrial genes <15%), but participant eligibility, sample-size justification, and MR sample selection are not reported. |
|  | c) | Describe measurement, quality control and selection of genetic variants | — | No measurement, quality control, or selection of genetic variants/instruments is reported in the current manuscript. |
|  | d) | For each exposure, outcome, and other relevant variables, describe methods of assessment and diagnostic criteria for diseases | 4-8 | Laboratory/analytical assessments are described for scRNA-seq processing, GO/KEGG analysis, qRT-PCR, and ELISA, but MR exposure/outcome assessment and disease diagnostic criteria are not explicitly reported. |
|  | e) | Provide details of ethics committee approval and participant informed consent, if relevant | 22 | Ethics information is incomplete: “Ethics declaration: Not available.” No committee name or informed-consent details are provided. |
| 5 | **Assumptions** | Explicitly state the three core IV assumptions for the main analysis (relevance, independence and exclusion restriction) as well assumptions for any additional or sensitivity analysis | — | The three core IV assumptions (relevance, independence, exclusion restriction) are not explicitly stated. |
| 6 | **Statistical methods: main analysis** | Describe statistical methods and statistics used | 4-8 | General statistical methods are described (Seurat workflow, Wilcoxon test, hypergeometric test with Benjamini-Hochberg correction, Student’s t test/Mann-Whitney U), but no MR-specific main-analysis statistics are reported. |
|  | a) | Describe how quantitative variables were handled in the analyses (i.e., scale, units, model) | 8 | Quantitative data are summarized as “mean ± SEM”; qRT-PCR relative expression is calculated with the 2^-ΔΔCt^ method; thresholds for cell filtering are also specified. |
|  | b) | Describe how genetic variants were handled in the analyses and, if applicable, how their weights were selected | — | No handling of genetic variants or weighting scheme is reported. |
|  | c) | Describe the MR estimator (e.g. two-stage least squares, Wald ratio) and related statistics. Detail the included covariates and, in case of two-sample MR, whether the same covariate set was used for adjustment in the two samples | — | No MR estimator (e.g., IVW, Wald ratio, MR-Egger, two-stage least squares) or MR covariate-adjustment strategy is reported. |
|  | d) | Explain how missing data were addressed | — | Missing-data handling is not described. |
|  | e) | If applicable, indicate how multiple testing was addressed | 5 | Multiple-testing control is mentioned only for enrichment analyses: “Benjamini-Hochberg correction” with adjusted P < 0.05; no MR-specific multiple-testing strategy is reported. |
| 7 | **Assessment of assumptions** | Describe any methods or prior knowledge used to assess the assumptions or justify their validity | — | No methods are reported to assess IV assumptions or justify their validity. |
| 8 | **Sensitivity analyses and additional analyses** | Describe any sensitivity analyses or additional analyses performed (e.g. comparison of effect estimates from different approaches, independent replication, bias analytic techniques, validation of instruments, simulations) | 18-19 | Additional validation is reported by qRT-PCR and ELISA, but no MR sensitivity analyses (e.g., weighted median, MR-Egger, MR-PRESSO, replication) are described. |
| 9 | **Software and pre-registration** |  |  |  |
|  | a) | Name statistical software and package(s), including version and settings used | 4-5, 8 | Software reported: Seurat v4.3.0 in R v4.2.0; clusterProfiler; R v4.2.0; GraphPad Prism v9.0; SPSS Statistics v26.0. |
|  | b) | State whether the study protocol and details were pre-registered (as well as when and where) | — | Pre-registration/protocol registration is not reported. |
|  | **RESULTS** |  |  |  |
| 10 | **Descriptive data** |  |  |  |
|  | a) | Report the numbers of individuals at each stage of included studies and reasons for exclusion. Consider use of a flow diagram | 4 | Quality-control thresholds are reported, but the numbers retained/excluded at each stage and reasons for exclusion are not fully quantified; no flow diagram is provided. |
|  | b) | Report summary statistics for phenotypic exposure(s), outcome(s), and other relevant variables (e.g. means, SDs, proportions) | 13, 18-19 | Summary statistics are reported for QC metrics and validation experiments (e.g., mean ± SEM expression values), but MR phenotypic exposure/outcome summaries are not reported. |
|  | c) | If the data sources include meta-analyses of previous studies, provide the assessments of heterogeneity across these studies | — | No meta-analysis heterogeneity assessment is reported. |
|  | d) | For two-sample MR:  i.  Provide justification of the similarity of the genetic variant-exposure associations between the exposure and outcome samples  ii.  Provide information on the number of individuals who overlap between the exposure and outcome studies | — | Two-sample MR sample comparability and sample overlap are not reported. |
| 11 | **Main results** |  |  |  |
|  | a) | Report the associations between genetic variant and exposure, and between genetic variant and outcome, preferably on an interpretable scale | 18 | Only a brief statement appears: “F11… consistent with the protective effect observed in Mendelian randomization analysis (OR=0.27).” Variant–exposure and variant–outcome associations are not otherwise reported. |
|  | b) | Report MR estimates of the relationship between exposure and outcome, and the measures of uncertainty from the MR analysis, on an interpretable scale, such as odds ratio or relative risk per SD difference | 18 | A single MR-related estimate is mentioned for F11 (OR=0.27), but full MR results and uncertainty measures are not systematically reported. |
|  | c) | If relevant, consider translating estimates of relative risk into absolute risk for a meaningful time period | — | Absolute-risk translation is not reported. |
|  | d) | Consider plots to visualize results (e.g. forest plot, scatterplot of associations between genetic variants and outcome versus between genetic variants and exposure) | 8-19 | Figures 1–9 visualize scRNA-seq and experimental validation results, but no MR-specific forest/scatter/funnel plots are provided. |
| 12 | **Assessment of assumptions** |  |  |  |
|  | a) | Report the assessment of the validity of the assumptions | — | Assessment of IV-assumption validity is not reported. |
|  | b) | Report any additional statistics (e.g., assessments of heterogeneity across genetic variants, such as *I^2^*, Q statistic or E-value) | — | No heterogeneity statistics across variants/instruments (e.g., Q, I²) or related MR diagnostics are reported. |
| 13 | **Sensitivity analyses and additional analyses** |  |  |  |
|  | a) | Report any sensitivity analyses to assess the robustness of the main results to violations of the assumptions | — | No MR robustness/sensitivity analyses to test assumption violations are reported. |
|  | b) | Report results from other sensitivity analyses or additional analyses | 18-19 | Additional analyses consist of qRT-PCR and ELISA validation of candidate genes; no additional MR sensitivity analyses are reported. |
|  | c) | Report any assessment of direction of causal relationship (e.g., bidirectional MR) | — | No assessment of causal direction (e.g., bidirectional MR, Steiger test) is reported. |
|  | d) | When relevant, report and compare with estimates from non-MR analyses | 18-21 | The manuscript discusses single-cell and experimental findings alongside limited MR references, but does not formally compare MR estimates with non-MR effect estimates. |
|  | e) | Consider additional plots to visualize results (e.g., leave-one-out analyses) | — | No leave-one-out or other MR-specific diagnostic plots are reported. |
|  | **DISCUSSION** |  |  |  |
| 14 | **Key results** | Summarize key results with reference to study objectives | 20-22 | Discussion and Conclusion summarize that the study provides a single-cell atlas, identifies cellular heterogeneity, and highlights F11, ACRP2, LEPR, and POSTN as potential therapeutic/prognostic biomarkers. |
| 15 | **Limitations** | Discuss limitations of the study, taking into account the validity of the IV assumptions, other sources of potential bias, and imprecision. Discuss both direction and magnitude of any potential bias and any efforts to address them | 22 | Limitations are discussed only for the single-cell component (“limited number of patients…”); limitations related to IV validity, pleiotropy, or MR bias are not discussed. |
| 16 | **Interpretation** |  |  |  |
|  | a) | Meaning: Give a cautious overall interpretation of results in the context of their limitations and in comparison with other studies | 20-22 | Overall interpretation is provided in the Discussion/Conclusion, but the MR component is only briefly referenced and is not interpreted with full methodological context. |
|  | b) | Mechanism: Discuss underlying biological mechanisms that could drive a potential causal relationship between the investigated exposure and the outcome, and whether the gene-environment equivalence assumption is reasonable. Use causal language carefully, clarifying that IV estimates may provide causal effects only under certain assumptions | 20-21 | Biological mechanisms are discussed for fibroblasts/CAF phenotypes, extracellular matrix remodeling, LEPR/POSTN signaling, and immune/metabolic pathways; causal language related to MR is not qualified by IV assumptions. |
|  | c) | Clinical relevance: Discuss whether the results have clinical or public policy relevance, and to what extent they inform effect sizes of possible interventions | 21-22 | Clinical relevance is discussed in terms of therapeutic targets, prognostic indicators, and precision medicine opportunities. |
| 17 | **Generalizability** | Discuss the generalizability of the study results (a) to other populations, (b) across other exposure periods/timings, and (c) across other levels of exposure | 22 | Generalizability is only partially addressed via the limitation that the single-cell analysis used a limited number of patients and may not represent all osteosarcoma subtypes/stages. |
|  | **OTHER INFORMATION** |  |  |  |
| 18 | **Funding** | Describe sources of funding and the role of funders in the present study and, if applicable, sources of funding for the databases and original study or studies on which the present study is based | 22 | Funding is reported: “This work was supported by the Natural Science Basic Research Program of Shaanxi Province (Grant No. 2022JM-516).” |
| 19 | **Data and data sharing** | Provide the data used to perform all analyses or report where and how the data can be accessed, and reference these sources in the article. Provide the statistical code needed to reproduce the results in the article, or report whether the code is publicly accessible and if so, where | 4, 22 | Data source is partly reported through GEO accessions “GSM8776323 and GSM4952363”; Data Availability states: “For further information, please contact the corresponding author.” Statistical code availability is not reported. |
| 20 | **Conflicts of Interest** | All authors should declare all potential conflicts of interest | 22-23 | Conflict-of-interest disclosure is incomplete: “Competing Interests Not available.” |

This checklist is copyrighted by the Equator Network under the Creative Commons Attribution 3.0 Unported (CC BY 3.0) license.

1. Skrivankova VW, Richmond RC, Woolf BAR, Yarmolinsky J, Davies NM, Swanson SA, et al. Strengthening the Reporting of Observational Studies in Epidemiology using Mendelian Randomization (STROBE-MR) Statement. JAMA. 2021;under review.

2. Skrivankova VW, Richmond RC, Woolf BAR, Davies NM, Swanson SA, VanderWeele TJ, et al. Strengthening the Reporting of Observational Studies in Epidemiology using Mendelian Randomisation (STROBE-MR): Explanation and Elaboration. BMJ. 2021;375:n2233.
